# Postpartum depression and its correlates: a cross-sectional study in southeast Iran

**DOI:** 10.1186/s12905-022-01978-6

**Published:** 2022-09-22

**Authors:** Mohammad Ali Zakeri, Somaye Khoram, Gholamreza Bazmandegan, Fatemeh Ghaedi-Heidari, Batol Talebi, Najmeh Ramezani, Fatemeh Ahmadi, Zahra Kamiab, Mahlagha Dehghan

**Affiliations:** 1 Non-Communicable Diseases Research Center, Rafsanjan University of Medical Sciences, Rafsanjan, Iran; 2 Social Determinants of Health Research Center, Rafsanjan University of Medical Sciences, Rafsanjan, Iran; 3 Department of Midwifery and Reproductive Health, Faculty of Nursing and Midwifery, Rafsanjan University of Medical Sciences, Rafsanjan, Iran; 4 Clinical Research Development Unit, Ali-Ibn Abi-Talib Hospital, Rafsanjan University of Medical Sciences, Rafsanjan, Iran; 5 Faculty of Nursing and Midwifery, Isfahan University of Medical Sciences, Isfahan, Iran; 6 Niknafs Hospital, Rafsanjan University of Medical Sciences, Rafsanjan, Iran; 7 Nursing Research Center, Kerman University of Medical Sciences, Kerman, Iran

**Keywords:** Pregnant, Mother, Anxiety, Stress, Postpartum depression

## Abstract

**Background:**

Postpartum depression has a negative impact on both infants and women. This study aimed to determine the correlates of postpartum depression in women in southern Iran.

**Methods:**

This cross-sectional study was performed on 186 mothers who had recently given birth to a baby. Data were collected using the demographic form, Quality of Prenatal Care Questionnaire, Edinburgh Postnatal Depression Scale (EPDS), Depression, Anxiety and Stress Scale—21 items (DASS-21) 3 days after delivery and EPDS and DASS-21 6 months after childbirth.

**Results:**

Postpartum depression (PPD) was 24.2% and 3.2% 3 days and 6 months after delivery, respectively. Anxiety, Prenatal Care Quality and educational level predicted 34.0% of the variance of PPD 3 days after delivery (R^2^ = 34.0%). Anxiety, type of delivery, and stress predicted 24% of the variance of PPD 6 months after delivery (R^2^ = 24.0%).

**Conclusions:**

With an increase in stress and anxiety and a reduction in the quality of prenatal care, the risk of postpartum depression increases. Therefore, attention to the quality of prenatal care and postpartum stress and anxiety should be carefully evaluated to prevent PPD. Psychological support and interventions are recommended to promote the mental health of women before and after childbirth.

## Introduction

Postpartum depression (PPD) is one of the most common disorders in women worldwide [1]. PPD is one of the most frequent complications, occurring in 10–15% of women after delivery [2]. PPD is also more prevalent in poor and middle-income countries than in rich ones. In Asian countries, the prevalence of this disorder varies from 7 to 33% [3]. In Iran, a study using the Edinburgh questionnaire showed that about 26% of the women who naturally conceived suffered from PPD [4]. The disorder has negative consequences for the mother and her child. In addition to having negative effects on a mother's quality of life [5]. PPD can increase a mother's chances of developing depression within 5 years after giving birth, so that Vigod et al. [6] reported that the risk of episodes of depression in women with PPD was twice as high in other women [6]. PPD is also harmful to the child and can delay his/her physical, social, and cognitive development [7].

Five common risk factors have been identified for PPD, including maternal characteristics, delivery characteristics, psychological factors, social support, and coping strategies [8]. Various studies have considered the socio-economic level of the mother, especially the low level of education and poor economic status, as factors related to PPD [9–11]. However, Cooper et al. [12] pointed out the significant heterogeneity of the impact of women's socioeconomic status on PPD [12]. A study in Japan found that women who did not receive adequate social support from their partners, even though they were socially supported by others, were significantly at risk for PPD [13]. Another study showed that in addition to insufficient social support, marital disharmony, depressive symptoms during pregnancy, a history of emotional problems, and long-term neonatal problems were among the predictors of PPD [14]. Guan et al. [8] showed that negative coping style was another factor associated with PPD [8].

In addition to the above factors, the quality of antenatal care can be associated with PPD [15]. Prenatal care is health care that a woman gets while pregnant. Women and infants who get proper prenatal care experience fewer unpleasant postpartum consequences [16]. Lua et al. [17] showed that the prevalence of PPD was 10.9% in women who got adequate prenatal care and 21.1% in women who did not get prenatal care [17]. A retrospective study showed that maternal and neonatal outcomes, including preterm delivery, placental abruption, PPD, low birth weight, neonatal respiratory distress syndrome, and intrauterine fetal demise, were significantly higher in depressed women during pregnancy who did not get adequate prenatal care than in women who got adequate prenatal care [18]. One study found that a well-perceived quality of care by women modulated and reduced PPD risk factors [15].

PPD is preventable in most cases, and early detection leads to easier treatment. On the other hand, a major part of prevention is to identify related risk factors. In addition, as depressed women are less likely to seek professional help, identifying risk factors for PPD provides an opportunity for health care providers to take preventive interventions for risky women at a timely date [19]. In addition, the prevalence of postpartum depression is related to socio-cultural factors, and risk factors are variable in different cultures [20]. Furthermore, few studies have examined the relationship between the quality of prenatal care and PPD. Therefore, the present study aimed to evaluate the factors associated with PPD, including prenatal care, in southeastern Iran. The following specific objectives were considered. (a) assessing the level of PPD, anxiety, stress and prenatal care quality, (b) assessing the association between demographic characteristics and PPD, (c) assessing the association between anxiety, stress and prenatal care quality with PPD, and (d) assessing the association of all important study variables with PPD using multivariate linear regression.

## Methods

### Study design and participants

This cross-sectional correlational study was performed to investigate the relationship between prenatal care, depression, anxiety, stress, and postpartum depression in women who have recently given birth to a baby. The research samples are women who gave birth (via vaginal delivery or cesarean section) 3 days ago. The sample size included 186 women who gave birth to a baby in the Nik-nafs maternity ward of Rafsanjan. The inclusion criteria were: (1) mothers aged 18 or above; (2) mothers without known psychological problems and disorders; (3) mothers without visual and auditory processing disorders. The exclusion criteria were: (1) existence of gynecologic diseases affecting the status of pregnancy, pregnancy results, and maternal and infant health, (2) termination of pregnancy due to preterm premature rupture of the membranes.

### Sample size and sampling

Based on studies by Izadirad et al. [21] to determine the relationship between health literacy and prenatal care adequacy index (r = 0.244) with 99% confidence and 90% test power, the sample size was considered to be 140 people according to the following formula. Concerning the conditions of mothers and the possibility of non-response, 200 questionnaires were distributed.

$$\upomega =\frac{1}{2}\mathit{Ln}\frac{1+r}{1-r}$$

$$n=\frac{{\left({Z}_{1-\frac{\alpha }{2}}+{Z}_{1-\beta }\right)}^{2}}{{(\omega )}^{2}}+3$$

Finally, 186 mothers completed the questionnaires.

### Measurement

#### Demographic information

Demographic information of the participants included age, Body Mass Index (BMI) of the mother, sex of the baby, type of delivery, previous delivery, number of deliveries, number of pregnancies, history of abortion, number of children, employment status, level of education, and income.

#### Edinburgh postnatal depression scale (EPDS)

This 10-item self-reported measure is designed to screen women for depressive symptoms during pregnancy and the postnatal period with scores from 0 to 3 (the maximum EPDS score is 30). The cut-off point remains at 13 or more, suggesting antenatal depressive symptoms. This questionnaire was developed by Cox et al. [22], and has been used in different countries to study postpartum depression [23]. Montazeri et al. [24] have used it in Iran and the internal correlation coefficient has been 0.80 [24]. In the present study, the reliability of the EPDS scale using Cronbach's alpha coefficient was 0.79 and 0.76 3 days and 6 months after delivery, respectively.

#### Depression, anxiety, stress scale (DASS-21)

The Depression, Anxiety, and Stress Scale (DASS-21), developed by Lovibond and Lovibond in 1995, was designed to assess the psychological constructs of depression, anxiety, and stress [25]. The scale consists of 21 items, including seven items for each of the three subscales of depression (7 items), anxiety (7 items) and stress (7 items) on a four-point Likert scale (never/low/medium/high). The lowest score is zero, and the highest score is three. The total score of each is obtained through the sum of the scores of the related items. The total score of the subscales should be doubled. Zakeri et al. [26] In Iran, Cronbach's alpha coefficient was reported to be 0.81, 0.74, and 0.78, for depression, anxiety, and stress, respectively, for Iranian version of DASS-21 [26]. In the present study, the reliability of the DASS-21 scale using Cronbach's alpha coefficient respectively was 0.75 and 0.80 for anxiety and stress 3 days after delivery. In addition, the reliability of the DASS-21 scale was 0.93 and 0.79 for anxiety and stress, respectively, 6 months after delivery. In the present study, we used two subscales of anxiety and stress.

#### Quality of prenatal care questionnaire (QPCQ)

This questionnaire was developed and validated by Sword et al. [27] in an Australian context to measure prenatal care quality. QPCQ includes 46 items with six subscales, including (1) Information sharing: focus on how prenatal care providers answer questions, keep information confidential, and ensure women understand reasons for tests (9 items), (2) Anticipatory Guidance: women should get enough information to make decisions (11 items), (3) Sufficient Time: the time prenatal care providers spend (5 items), (4) Approachability: the health care provider’s approachability (4 items), (5) Availability: the availability of the clinic/office staff or prenatal care provider in 5 items, and (6) Support and Respect: respect and support by prenatal care providers in 12 items. The items are on a five-point Likert scale (from strongly disagree = 1 to strongly agree = 5). Items 8, 15, 23, 28, and 40 are scored reversely. The total score of QPCQ (46 questions) ranges from 46 to 230, with a higher score reflecting a higher quality of prenatal care. The QPCQ is a valid and reliable measure of the overall quality of prenatal care [27]. In the present study, the validity of this questionnaire was obtained by using face and content validities. We used internal consistency and test–retest for the QPCQ to assess reliability. The internal consistency was good (α = 0.94) and the Intraclass correlation coefficient was 0.47 [28].

#### Data collection and statistical analysis

The researcher referred to the research settings and started sampling in Niknafs maternity ward after obtaining the necessary permission. Thus, the demographic information form, QPCQ, EPDS, and DASS-21 questionnaire (subscales of anxiety and stress) were distributed among the eligible samples, who answered the questionnaires in the presence of the researcher (face-to-face). In addition, The EPDS and DASS-21 were completed and evaluated 6 months after delivery. EPDS and DASS-21 were assessed with a telephone interview 6 months after delivery. Two hundred questionnaires were distributed over 5 months (October 2019 to February 2020), and 186 copies were returned (response rate: 93%). Finally, 186 samples were included in the study. No questionnaires were excluded from the study. The data were then analyzed by SPSS 22 and significance level of 0.05 was considered. Descriptive statistics (frequency, percentage, mean and standard deviation) were used to describe the information. Pearson correlation coefficients were used to determine the relationship between the quantitative variables of the study. The independent *t*-test, Mann–Whitney U, ANOVA, and Kruskal–Wallis tests (considering the normality of the data) were used to determine PPD according to the qualitative variables of the study. If the variable was normally distributed, an independent t-test was used to compare PPD according to two groups, and an ANOVA test was used to compare PPD according three or more groups. If the variable was not normally distributed, the Mann–Whitney U test was used to compare PPD according to two groups, and the Kruskal–Wallis test was used to compare PPD according to three or more groups. Multivariate linear regression was used to identify the PPD determinants. We used multiple linear regression to estimate the relationship between the independent variables of the present study and the dependent variable of PPD (after delivery and 6 months after delivery). A significance level of 0.05 was considered.

## Results

The mean age of the participants was 29.24 ± 5.89 years. 76.90% of the participants underwent cesarean section, 37.10% of them were nulliparous, 89.20% were housewives, 45.20% had a diploma, 33.30% gave birth to one baby, and 81.20% had no history of abortion (Table 1).

**Table 1:** Participants’ demographic characteristics and information and their association with PPD (After delivery and 6 month after delivery) among the participants (N = 186)

Variables	Mean (SD)	PPD (after delivery)		PPD (6 month after delivery)	
		Pearson correlation coefficient	*P* value	Pearson correlation coefficient	*P* value
Age of mother (year)	29.24 (5.89)	0.11	*P* = 0.88	0.00	*P* = 0.92
BMI of mother	29.25 (4.74)	− 0.00	*P* = 0.93	− 0.05	*P* = 0.43

	*N* (%)	Statistical test	*P* value	Statistical test	*P* value
*Sex of the baby*					
Boy	93 (50.00)	*t* = 1.01	*P* = 0.31	*t* = − 1.05	*P* = 0.29
Girl	93 (50.00)				
*Type of delivery*					
Vaginal	43 (23.10)	*t* = − 2.19	*P* = 0.030*	*t* = 1.72	*P* = 0.08
Cesarean section	143 (76.90)				
*Previous delivery*					
Vaginal	41 (22.00)				
Cesarean section	76 (40.90)	H = 2.44	*P* = 0.29	H = 4.70	*P* = 0.09
None	69 (37.10)				
*Number of deliveries*					
1	62 (33.30)				
2	60 (32.30)	F = 1.22	*P* = 0.30	F = 1.61	*P* = 0.18
3	40 (21.50)				
4 ≤	24 (12.90)				
*Number of pregnancies*					
1	68 (36.60)				
2	70 (37.60)	F = 0.02	*P* = 0.98	F = 1.55	*P* = 0.16
3 ≤	48 (25.80)				
*Abortion*					
Yes	35 (18.80)	*t* = 1.01	*P* = 0.31	*t* = 0.84	*P* = 0.40
No	151 (81.20)				
*Number of children*					
1	69 (37.10)				
2	71 (38.20)	F = 0.08	*P* = 0.91	F = 1.65	*P* = 0.19
3 ≤	46 (24.70)				
*Employment status*					
Employed	20 (10.8)	Z = − 0.42	*P* = 0.67	Z = − 1.49	*P* = 0.13
Housewife	166 (89.2)				
*Educational level*					
< Diploma	41 (22.0)	H = 8.88	*P* = 0.012*	H = 5.10	*P* = 0.07
Diploma	84 (45.2)				
Academic	61 (32.8)				
*Income (million riyal)*					
< 0.5	99 (53.2)				
0.5–1	47 (25.3)	H = 1.14	*P* = 0.56	H = 1.58	*P* = 0.45
> 1	40 (21.5)				

Data were presented numerically (%). The sample consisted of 186 participants with mean age of 29.24 ± 5.89 years. Z = Mann Whitney u test; H = Kruskal Wallis test; F = ANOVA test. *p < 0.05. PPD: Postpartum depression

The mean score of prenatal care quality was 164.96 ± 22.10, which was greater than the midpoint of the questionnaire (score = 115). The mean score of PPD was 8.81 ± 4.17 after delivery, which was lower than the midpoint of the questionnaire (score = 13). The mean score of PPD was 2.48 ± 3.13 6 months after delivery, which was much lower than the midpoint of the questionnaire (score = 13). The mean scores of anxiety and stress were 7.62 ± 6.95 and 11.72 ± 8.83, respectively, after delivery. The mean scores of anxiety and stress were 1.09 ± 4.24 and 1.52 ± 3.71, respectively, 6 months after delivery. Based on the score of the EPDS, 45 mothers (24.2%) had postpartum depression after delivery, and six mothers (3.2%) had postpartum depression 6 months after delivery (Table 2).

**Table 2:** Distribution of prenatal care quality, PPD, anxiety, stress among participants (N = 186)

Variables	N (%)	Mean	SD	Min	Max
PPD (after delivery)	186 (100)	8.81	4.17	0	25
PPD (6 months after delivery)	186 (100)	2.48	3.13	0	16
Anxiety (after delivery)	186 (100)	7.62	6.95	0	30
Anxiety (6 months after delivery)	186 (100)	1.09	4.24	0	28
Stress (after delivery)	186 (100)	11.72	8.83	0	38
Stress (6 months after delivery)	186 (100)	1.52	3.71	0	22
Total score prenatal care quality	186 (100)	164.96	22.01	89.00	219.00
Information sharing	186 (100)	34.63	5.02	20.00	45.00
Anticipatory guidance	186 (100)	39.65	6.90	18.00	55.00
Sufficient time	186 (100)	18.35	2.48	7.00	24.00
Approachability	186 (100)	9.75	3.47	4.00	20.00
Availability	186 (100)	17.64	3.59	7.00	25.00
Support and respect	186 (100)	44.91	6.65	22.00	60.00

Data were presented as number, mean, standard deviation, minimum and maximum for each variable

*PPD* postpartum depression

The PPD score was significantly associated with type of delivery (*P* = 0.03) and educational level (*P* = 0.01). No significant correlation was found between PPD score, demographic and clinical information variables 6 months after delivery (Table 1).

Pearson correlation coefficient between PPD after delivery and PPD 6 months after delivery (r = 0.16, *p* = 0.03). A significant association was observed between PPD after delivery, anxiety after delivery (*p* < 0.001), stress after delivery (*p* < 0.001) and stress 6 months after delivery (*p* = 0.004). A significant and strong association was observed between PPD 6 months after delivery, anxiety (*p* < 0.001) and stress 6 months after delivery (*p* = 0.006). Stress variable after delivery and 6 months later had a significant association with PPD after delivery and PPD 6 months after delivery (*p* < 0.05). A negative significant association was observed between prenatal care quality and PPD after delivery (r =  − 0.26, *p* < 0.001), anxiety (r =  − 0.16, *p* = 0.02), and stress after delivery (r =  − 0.20, *p* = 0.004).

For further analysis, the multivariate regression with backward method was conducted. All variables with a p-value < 0.2 were included in the multivariate regression model. Anxiety after delivery, prenatal care quality and educational level predict 34% of the variance of PPD after delivery (R2 = 34%), and the best predictor is anxiety (*p* < 0.001). Anxiety 6 months after delivery, type of delivery, and stress after delivery predict 24% of the variance of PPD 6 months after delivery (R2 = 24%), and the best predictor is anxiety 6 months after delivery (*p* < 0.001) (Table 3).

**Table 3:** Regression analysis summary for anxiety, stress and prenatal care quality variables predicting PPD (after delivery and 6 months after delivery) (N = 186)

Variables	B	SE	β	T	p	95%CI lower	95%CI upper	Adjusted R2
PPD (after delivery)								34%
(Constant)	13.92	2.24		6.19	0.000	9.48	18.35	
Anxiety (after delivery)	0.14	0.05	0.24	2.90	0.004	0.04	0.24	
Prenatal care quality	− 0.03	0.01	− 0.18	− 2.98	0.003	− 0.05	− 0.01	
Educational level	− 0.85	0.34	− 0.15	− 2.47	0.01	− 1.53	− 0.17	
PPD (6 months after delivery)								24%
(Constant)	3.64	0.88		4.13	0.000	1.90	5.38	
Anxiety (6 months after delivery)	0.16	0.05	0.22	3.35	0.001	0.07	0.26	
Type of delivery	− 1.19	0.47	− 0.16	− 2.50	0.01	− 2.14	− 0.25	
Stress (after delivery)	0.05	0.02	0.14	2.24	0.03	0.00	0.09	

*PPD* postpartum depression; *B* the unstandardized beta; *SE B* the standard error for the unstandardized beta; *β* the standardized beta; *t* the *t* test statistic; *p* the probability value and *CI* confidence interval

## Discussion

The results of the present study showed that approximately one in four women suffered from PPD 3 days after childbirth, a frequency that decreased 6 months after delivery, so that only 3.2% of the women had PPD 6 months after delivery. The decreasing frequency of patients with PPD after 6 months in the present study is consistent with scientific evidence because hormonal fluctuations, especially in the first week after delivery, increase the chances of mood swings and depression more than other times [2, 29]. In addition, a study in Sri Lanka used EDPS with a cut-off point of 9 and showed that the prevalence of PPD was 15.5% 10 days after delivery and 7.8% four weeks after delivery [2]. The prevalence of PPD has been reported with high variability, with 8% in Europe, 16% in Asia and 26% in the Middle East [30]. High variability in the prevalence of PPD in studies can have various causes, including cultural differences [20], differences in study time, PPD assessment method, cut-off point for EDPS, sample size, and study methodology. The present study examined PPD in women using EDPS and a cut-off point of 13. Anokye et al. (2018) studied Ghanaian women 12 months after delivery using the Patient Health Questionnaire (PHQ) and showed that the prevalence of PPD was 7% [31]. Using EDPS and a cut-off point of 13, Asaye et al. [32] showed that the prevalence of PPD in Ethiopian women was 25% six weeks after delivery. Using EDPS and a cut-off point of 10, Peng et al. [33] reported that the prevalence of PPD in Chinese women was 11.6% from birth to six weeks after delivery [33].

The results of the study using a multivariate regression test showed that anxiety, quality of prenatal care, and education level were significant predictors of PPD 3 days after delivery. Therefore, the risk of developing PPD increased with an increase in the anxiety score and a decrease in the perceived quality of prenatal care and education level. Consistent with the present study, one study showed that PPD was comorbid with anxiety in 75% of cases, and anxiety was a significant variable for PPD [34]. One study also showed that the higher the perceived quality of care by women, the lower the risk of PPD [15]. The results of several studies showed that the lower the education level of the individual, the higher the risk of PPD [35–37], which is confirmed by the results of the present study. This result may be justifiable because low levels of education are associated with low incomes, and the poorer a person's economic situation, the greater the risk of PPD [38].

The results of the present study also showed that in addition to anxiety, stress and type of delivery were significant variables to predict the risk of PPD 6 months after delivery, such that the higher scores of stress, anxiety, and cesarean delivery put women at greater risk for PPD. In line with the present study, Caparros-Gonzalez et al. [39] showed that high stress, especially during pregnancy, could predict the likelihood of developing PPD [39]. Another study found that the more a person experienced stress in his/her life, the more likely he/she was to develop PPD [40]. The results of a meta-analysis showed a significant relationship between cesarean section and PPD. Cesarean section increases the risk of PPD [41], which is in line with the results of the present study. This study has several limitations. First, the study design is cross-sectional and no causal relationship can be inferred between the studied factors and PPD based on its results. Second, self-reported questionnaires were used in the present study, which could affect the study results. Third, EDPS is a screening tool rather than a diagnostic tool that can affect results.

## Conclusion

According to the results of the study, the prevalence of PPD decreased from 24.2% 3 days after delivery to 3.2% 6 months after delivery. High stress and anxiety, poor quality of prenatal care, cesarean section and low level of education were risk factors for PPD. Perceived quality of prenatal care, stress, and anxiety are changeable variables. Therefore, according to the results of the study, it is suggested that appropriate psychological support and interventions be designed and implemented for mothers before and after childbirth to promote their mental health. In addition, it is recommended that healthcare providers provide women with the necessary quality care during pregnancy. Since the present study was performed using EDPS as a PPD screening tool, it is suggested that diagnostic tools and interviews be used in future studies to evaluate PPD.

## Data Availability

The dataset generated and/or analysed during the current study are not publicly available due to promises of participant anonymity and confidentiality but are available from the corresponding author on reasonable request.

## Abbreviations

BMI: Body Mass Index
DASS-21: Depression, anxiety and stress scale—21
EPDS: Edinburgh postnatal depression scale
QPCQ: Quality of prenatal care questionnaire
PPD: Postpartum depression
